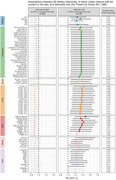# Dietary exposure to chemical contaminants and risk of dementia in older persons

**DOI:** 10.1002/alz.089322

**Published:** 2025-01-09

**Authors:** Sophie Lefèvre‐Arbogast, Pauline Duquesne, Catherine Helmer, Véronique Sirot, Cécilia Samieri

**Affiliations:** ^1^ Bordeaux Population Health Research Center, Inserm U1219, University of Bordeaux, Bordeaux France; ^2^ ANSES, Direction de l’évaluation des risques, Maisons‐Alfort France

## Abstract

**Background:**

Diet is a primary source of exposure to chemicals in the general population. Dietary contaminants originate from food production (e.g.pesticides) or from environmental contamination by industrial (polychlorinated biphenyls [PCB]) or naturally‐occurring substances (metals). Many are present at non‐negligible levels in human biofluids, and some were found in the human brain. Since many chemicals are lipophilic, the dietary composition, especially the fat content, likely modifies their toxicokinetics. We aimed to explore the link between dietary exposure to multiple chemicals and dementia risk in older persons, considering effect modification by dietary fat content.

**Method:**

We included 1,288 non‐demented participants from the French Three‐City cohort who completed a food survey (2001‐2002) and were followed for incident dementia. Dietary exposure to 167 chemicals (covering 9 families) was assessed by combining individual food intakes with food chemical content measured in the French second Total Diet Study. We assessed the relation of each individual chemical with dementia risk using Cox proportional hazard models adjusted for age, sex, education, ApoE‐ε4 and Mediterranean diet adherence and exploring effect modification by high‐fat diet content (defined as >35% of energy from fat). We subsequently considered all chemicals simultaneously and performed chemical variable selection using elastic‐net regularization.

**Result:**

Participants were 76 years‐old on average at baseline; 62% were women; 30% reported a high‐fat diet. Over a median follow‐up of 10 years, 314 individuals developed dementia. No chemical was associated with dementia risk in the whole population. However, having a diet with higher fat content was a strong effect modifier for 82 chemicals (FDR‐corrected p<0.05 for interaction tests). Higher intakes of these chemicals were significantly associated to higher dementia risk among high‐fat diet consumers only. The strongest hazard ratios (HR>1.40 for one SD‐increase in intake) were observed for contaminants provided by seafood and meat, including the brominated flame retardant hexabromocyclododecane‐alpha (HBCDD‐α), perfluorooctane sulfonate (PFOS) and PCB‐77. Among high‐fat diet consumers, the elastic‐net identified 33 chemicals associated with dementia risk, with PFOS and nitrites ranking as primary chemicals.

**Conclusion:**

In our study, dietary exposure to specific chemicals, was associated with higher dementia risk among older persons consuming >35% of energy from fat in their diet.